# Neurovascular Network Explorer 2.0: A Database of 2-Photon Single-Vessel Diameter Measurements from Mouse SI Cortex in Response To Optogenetic Stimulation

**DOI:** 10.3389/fninf.2017.00004

**Published:** 2017-02-01

**Authors:** Hana Uhlirova, Peifang Tian, Kıvılcım Kılıç, Martin Thunemann, Vishnu B. Sridhar, Hauke Bartsch, Anders M. Dale, Anna Devor, Payam A. Saisan

**Affiliations:** ^1^Department of Radiology, University of CaliforniaSan Diego, La Jolla, CA, USA; ^2^Central European Institute of Technology, Brno University of TechnologyBrno, Czechia; ^3^Institute of Physical Engineering, Faculty of Mechanical Engineering, Brno University of TechnologyBrno, Czechia; ^4^Department of Neurosciences, University of CaliforniaSan Diego, La Jolla, CA, USA; ^5^Department of Physics, John Carroll University, University HeightsOH, USA; ^6^Bioengineering Undergraduate Program, University of CaliforniaSan Diego, La Jolla, CA, USA; ^7^Martinos Center for Biomedical Imaging, Massachusetts General Hospital, Harvard Medical SchoolCharlestown, MA, USA

**Keywords:** neuroinformatics, graphical user interface, MATLAB, arterioles, blood flow, hemodynamics, cerebrovascular circulation, inhibitory neurons

## Introduction

Sharing of experimental data is of critical importance in neuroscience allowing a close inspection by the research community and facilitating the use of experimental data for modeling. However, with a few exceptions, data from individual studies conducted by regular size neuroscience laboratories are not shared. Previously, we provided an example of seamless and low-cost solution for sharing of such data. Specifically, we created a MATLAB® based Graphical User Interface (GUI) engine, which we called Neurovascular Network Explorer 1.0 (NNE 1.0), to interact with a database of 2-photon measurements of sensory stimulus-induced diameter changes of rat cortical arterioles *in vivo* (Sridhar et al., 2014). NNE 1.0 and the associated database can be downloaded by the user from our academic website (http://nil.ucsd.edu/data/NNE/NNE1_Tian/). The GUI runs either as a MATLAB script or as a standalone program on a Windows platform. It allows browsing the database according to parameters specified by the user, simple manipulation, and visualization of the retrieved records (such as averaging and peak-normalization), and export of the results. The same website provides the NNE 1.0 source code. With this source code, the user can database their own experimental results, given the appropriate data structure and naming conventions, and thus share their data in a user-friendly format with other investigators.

Here, we present a novel database containing 2-photon data from our recently published experimental study (Uhlirova et al., 2016a) and a second generation of our GUI-based software engine that we call NNE 2.0. The data, GUI, and source code are freely available for download from our academic website (http://nil.ucsd.edu/data/NNE/NNE2_HDbase_v1.0/). The database contains 2-photon measurements of arteriolar diameter changes in response to selective optogenetic (OG) activation of cortical inhibitory neurons (INs). All measurements were performed in the mouse primary sensory cortex (SI). In addition to all functionalities of NNE 1.0, NNE 2.0 supports 3D visualization of the structural vascular data and localization of individual measurements within the structural vascular network. This new feature can be utilized by the user for computational reconstruction (“graphing”) of the microvascular network, similar to what was done in our published studies (Sakadzic et al., 2010, 2014; Gagnon et al., 2015). Such reconstructions can provide a realistic foundation for bottom-up modeling of the vascular/hemodynamic responses, which are important for understanding cerebral blood flow regulation and physiological underpinning of non-invasive imaging signals (Gagnon et al., 2015; Uhlirova et al., 2016b).

## Data source

On the level of cortical arterioles, the hemodynamic response to a sensory stimulus is composed of a combination of dilatory and constrictive phases with the relative strength of vasoconstriction co-varying with that of neuronal hyperpolarization (i.e., inhibition; Devor et al., 2007, 2008; Tian et al., 2010; Nizar et al., 2013). Recently, we and others provided direct evidence that activation of INs can regulate arteriolar diameters *in vivo* (Anenberg et al., 2015; Uhlirova et al., 2016a). In our study, we used *in vivo* 2-photon measurements of arteriolar diameters in the mouse cerebral cortex in response to selective OG activation of INs (Uhlirova et al., 2016a). Our results confirmed the ability of INs to drive the biphasic arteriolar response and serve as the primary mediators of vasoconstriction in cortical arterioles under normal conditions. Here, we present an interactive database corresponding to this study. All experimental procedures in the original study were performed in accordance with the guidelines established by the UCSD Institutional Animal Care and Use Committee (IACUC).

## Database structure and naming conventions

### Overview

The principal database entry is a temporal profile (i.e., a time-course) of single-vessel diameter change measured at a specific location along an arteriolar tree in the mouse primary somatosensory cortex. In addition to the diameter time-course, each database entry holds an array of descriptive parameters, such as the cortical depth and branching order (BO) (Table 1; see “*The vdb_gnu matrix and parameters”* below).

**Table 1:** **The vdb_gnu parameters**.

	**Parameter name**	**Description**
vdb_gnu {i,1}	“Date”	Unique subject identifier corresponding to the calendar date of the experiment.
vdb_gnu {i,2}	“Set”	Arteriolar tree identifier; unique for subject.
vdb_gnu {i,3}	“Time”	A time vector for the *Timecourse*, see below. *Time* is in seconds relative to the stimulus onset.
vdb_gnu {i,4}	“Timecourse”	Arteriolar diameter change as a function of time. *Timecourse* is in percent change from the baseline. *Time* and *Timecourse* have the same size.
vdb_gnu {i,5}	“Normalized timecourse”	*Timecourse* normalized by the peak amplitude.
vdb_gnu {i,6}	“Branching order”	Branching order at the measurement location. The possibilities are: surface arteriole, diving trunk, 1^st^ order branches, and higher order branches^*^.
vdb_gnu {i,7}	“X-axis intercept”	The estimated onset of dilation. The onset was quantified by fitting a straight line to the rising slope of the diameter increase between 20 and 80% of the peak amplitude and calculating an intercept with the pre-stimulus baseline.
vdb_gnu {i,8}	“Time of peak”	The time when vessel diameter reaches its peak dilation.
vdb_gnu {i,9}	“Depth”	The approximate depth of the measurement below the cortical surface.
vdb_gnu {i,10}	“Baseline diameter”	Vessel diameter prior to stimulus onset.
vdb_gnu {i,11}	“Peak dilation amplitude”	Peak dilation expressed as percent change from the baseline.
vdb_gnu {i,12}	“Stimulus”	Type of stimulus: optogenetic (“OG”).
vdb_gnu {i,15}	“Run number”	Unique identifier of the measurement per subject; reflects the sequential order of measurements across locations within the vascular network.
vdb_gnu {i,16}	“Ref image pointer”	The pointer to the reference image of the horizontal (XY) plane of the measurement. The measured vessel is in the center of the image.
vdb_gnu {i,17}	“Z-stack pointer”	The pointer to the associated structural 3D volume.
vdb_gnu {i,19}	“Z-stack index”	Index of a frame in the z-stack matched to the reference image.
vdb_gnu {i,21}	“Ref image umpp”	Scale for the reference image, μ/pixel.
vdb_gnu {i,22}	“Z-stack umpp”	Scale for the XY planes of the 3D stack, μ/pixel.
vdb_gnu {i,23}	“Map pointer”	The pointer to a low-magnification image of the surface vasculature; unique for a subject. Locations of the initial diving segments for imaged arteriolar tree are labeled by their *Tree ID*.
vdb_gnu {i,24}	“Z-stack step”	Vertical distance between two consecutive frames in the 3D image stack.

* *The higher order branches include 2^nd^, 3^rd^, and 4^th^ order branches. The GUI lumps these as a single category. However, the user can access individual categories when working in Matlab*.

**Figure 1:**
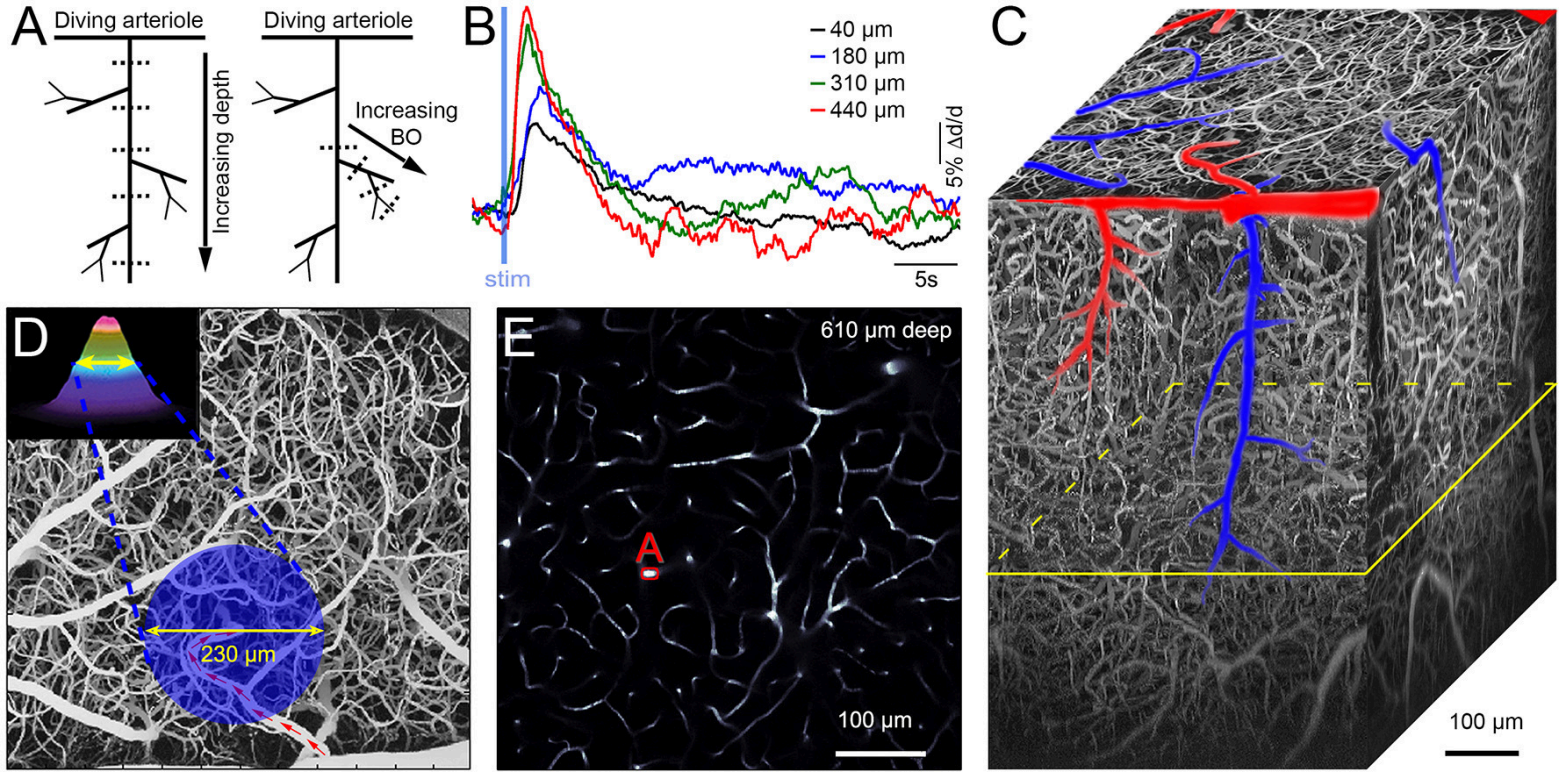
**(A)** Schematic illustration of a single diving and branching arteriolar tree. Measurement locations are indicated by dotted lines (representing 2-photon line-scan across the imaged vessel). The left panel illustrates the idea of multiple measurements at different depths along the diving arteriolar trunk. The right panel shows a number of measurements along arteriolar segments of increasing BO starting from the upstream diving trunk. **(B)** An example family of dilation time-courses acquired at different depths along an individual diving arteriolar trunk in response to OG stimulus. **(C)** Three-dimensional projection of an image stack (500 2-photon XY-planes spaced at 2 μm along the Z-axis). The vasculature is visualized by intravascular injection of Fluorescein isothiocyanate (FITC)-labeled dextran. **(D)**. A weighted maximum intensity projection (MIP) of the image stack shown in **(C)**. Schematic illustration of the OG beam, centered on the initial diving segment of a penetrating arteriole, is overlaid on the MIP. Blood flow direction in the parent surface arteriole is labeled by red arrows. **(E)** A single image plane from **(C)** 610 μm below the cortical surface; its location within the stack is indicated in **(C)** by the yellow parallelogram. The label “**(A)**” indicates a cross section of a penetrating arteriole. The OG beam in **(D)** is centered on the initial diving segment of the same arteriole.

Each arteriolar tree is composed of the diving trunk and side branches, such that the obtained dilation time-courses can be classified on the basis of the depth below the cortical surface (“cortical depth”) and BO, as schematically illustrated in Figure 1A. Since each time-course entry reflects a response to stimulation, it can be viewed as “functional” data. This is in contrast to 2D or 3D images of vasculature that are anatomical in essence, and will be referred to as “structural” data. Figure 1B illustrates a family of time-courses obtained along a single diving arteriolar trunk. These measurements were acquired sequentially while repeating the same OG stimulus.

We used transgenic [VGAT-ChR2(H134R)-EYFP] mice expressing the OG actuator ChR2 in all INs (Zhao et al., 2011). The OG stimulus consisted of a double pulse of blue laser light for the total stimulus duration of 450 ms. The stimulus was delivered through the objective using a 473-nm cylinder-shaped laser beam ~230 μm in diameter that is comparable to the size of a cortical column (Figure 1D). Additional details on the OG stimulus are available in the original publication (Uhlirova et al., 2016a). Only a single OG trial was presented at each measurement location, and the database entry corresponds to a single-trial time-course. The database contains 305 measurements (time-courses) along 93 arteriolar trees (217 and 88 measurements along diving trunks and branches, respectively) in 17 subjects at depths from 30 to 560 μm.

### The vdb_gnu matrix and parameters

As with the NNE 1.0 (Sridhar et al., 2014), the current database is organized as a MATLAB structure that we call “vascular database” (vdb_gnu) matrix. The first row in the matrix vdb_gnu (vdb_gnu{1,:}) contains names of parameters in each column (Table 1). Each of the remaining rows contains entries for each of these parameters for a particular measurement. This vdb_gnu has total of 306 rows and 24 columns (i.e., 305 individual time-course measurements).

The main innovation of NNE 2.0 compared to NNE 1.0 (Sridhar et al., 2014) is the availability of the structural data—3D image stacks and 2D reference images—that provide the exact location of the functional measurement within the corresponding vascular network (Figure 1C). The vasculature is visible due to the presence of a (green) fluorescent agent Fluorescein isothiocyanate (FITC)-labeled dextran in the blood stream (i.e., only inside the vasculature, Figures 1C–E).

A 3D image stack is a structural volume of vasculature composed of sequential horizontal (XY) 2-photon image planes spaced a few microns apart along the cortical depth axis (Z). For each subject, image stacks were acquired at the end of the experiment. Image stacks can be utilized by the user for computational reconstruction of the vascular architecture using segmentation algorithms published elsewhere (Hirsch et al., 2012; Blinder et al., 2013); for a recent review see Gagnon et al. (2016).

A reference image is a 2D image of the exact XY plane where the time-course measurement was obtained. The reference image was acquired immediately after the time-course measurement. The reference image can be matched to one of the images in the corresponding stack providing a possibility for registration of the functional measurement within the 3D structural volume (see Supplementary Materials, USER GUIDE).

Multiple database entries (vdb_gnu{i,:}), each corresponding to a dilation time-course from a different measurement location, can have the same corresponding image stack (coded by the “Z-stack pointer,” Table 1). This occurs when measurements belong to the same arteriolar tree (the same “Set”). It can also occur for measurements from different trees when multiple diving arterioles are captured within the same image stack. There may be more than one image stack per subject (a given mouse). This happens when two or more imaged arteriolar trees are separated in the XY plane further than what can be captured within the field of view of a high-magnification objective. To help the user orient in space, we provide a subject-specific low-magnification image of the surface vasculature within the exposed “cortical window” (“Map pointer,” Table 1). The user can recognize the location of each image stack by matching the surface vasculature of that stack to the low-resolution map. The initial diving segment for each imaged arteriole is labeled on this map by a number corresponding to its “Set” value.

In most cases, the plane of the cortical surface did not exactly match the XY imaging plane. As a result, image stacks are titled relative to the cortical surface. The “Depth” parameter (Table 1) is an approximate depth below the cortical surface that was manually entered by the researcher during the data acquisition. The GUI uses this information and the reference image to find and display the closest XY plane from the image stack and its depth within the stack. To further refine estimation of the depth, one can resample the image stack to align the XY plane with the cortical surface using algorithms available elsewhere. Example code has been made available on the Resources page of our academic website http://nil.ucsd.edu, under “Two-photon imaging.”

### NNE 2.0 GUI: advanced features and benefits

NNE 2.0 GUI was developed in MATLAB using NNE 1.0 architecture as a foundation (Sridhar et al., 2014). Similar to NNE 1.0, the NNE 2.0 allows browsing the database according to parameters specified by the user, manipulation and visualization of the retrieved records, and export of the results. The database, NNE 2.0 GUI, and its source code can be downloaded from our academic website (http://nil.ucsd.edu/data/NNE/NNE2_HDbase_v1.0/). The Installation_readme.txt and USER GUIDE documents (see Supplementary Materials) provide step-by-step instructions for the installation and operation procedures, respectively.

The primary architectural point of departure for NNE 2.0 from NNE 1.0 is in the way the image stacks and reference images are organized, accessed, and visualized. These structural data associated with each time-course entry (for outliers see Supplementary Materials, USER GUIDE) add a substantially large component to each data record. In NNE 1.0, the entire database was contained within one primary.mat file. In NNE 2.0, on the other hand, the presence of the structural data necessitated addition of external directories. Two such directories exist as separate entities holding image stacks (hana_stk directory) and reference images (hana_refs directory). In this way, the vdb_gnu.mat file is kept small as it does not include any images but references to the data within these two directories using MATLAB pointers (“Z-stack pointer” and “Ref image pointer,” Table 1).

This architecture allows a seamless and efficient vehicle to access the additional data components both for native MATLAB environment (where the user loads the vdb_gnu matrix directly into MATLAB) as well as for the standalone GUI program (running under any modern Windows operating system, Windows 7 or newer). One additional benefit of this organization is that users not interested in the structural data do not have to download the auxiliary image directories. When these directories are not present, the user will be able to proceed throughout the GUI and export the time-course data (see Supplementary Materials, USER GUIDE).

To display the structural data, NNE 2.0 includes a number of useful and powerful data visualization and user-interaction features within a dedicated GUI panel (see Supplementary Materials, USER GUIDE), which can be utilized as a reusable template. Examples are (i) the ability to choose time-courses on the fly from a displayed set, without disrupting all other processes, and (ii) the ability to call sub-routines to display the image data frame-to-frame, similar in its perceptual effect to a movie viewing tool, without requiring separate external programs. The underlying routines are modular and thus can be reused for building similar interactive data visualization panels.

## Conclusions and future development

Despite the growing awareness of the need for neuroinformatics and clear utility of data repositories supported by large consortia, the overwhelming majority of data from regular-sized neuroscience laboratories are currently not being shared. Each laboratory uses different (and often custom) data formats and conventions preventing meaningful data sharing. In addition, regular-sized neuroscience laboratories rarely have a dedicated budget for informatics.

The present article provides a practical example of circumventing these obstacles. Our GUI offers an intuitive engine to familiarize the user with the database visualizing functional and structural data. After exporting the data in MATLAB or Excel formats, the user can compare the output of their own data analysis software (e.g., a time-course plot) with that of the GUI to confirm their understanding of the data. Given that virtually every academic institution has MATLAB license for students and faculty, this also is an affordable solution.

The modular software organization of the GUI allows addition of new types of data, similar to the way we were able to add the structural image data. Further, the MATLAB/Excel based export format enables easy interface with post-database processing plugins to support segmentation/graphing, motion correction, additional visualization routines, and more.

Our GUI tool is by no means in competition with more advanced and complex GUI based tools or databasing systems, as it is constrained to the building blocks provided by MATLAB. Rather, it should be viewed as an efficient and low cost data sharing solution that can be easily adopted by a regular-size neuroscience laboratory with no need for dedicated informatics budget. Given the popularity of MATLAB, the main advantage of our solution is the ease and speed with which users can learn and create their own databasing/visualization tools working with this type of data.

Future updates to this database will be deposited in the same repository (http://nil.ucsd.edu/data/NNE/), and the relevant information will be published as a General Commentary linked on the Frontiers website to this Data Report.

## Author contributions

HU: conceptual design, data analysis, designing the USER GUIDE, critically revising the manuscript; PT, KK, MT, VS: data analysis, critically revising the manuscript; HB, AMD: conceptual design, critically revising the manuscript; AD: conceptual design, drafting, and revising the manuscript, critically revising the USER GUIDE; PS: software design, critically revising the manuscript and the USER GUIDE; All authors approved the final version of the manuscript to be published and agree to be accountable for all aspects of the work in ensuring that questions related to the accuracy or integrity of any part of the work are appropriately investigated and resolved.

## Funding

We gratefully acknowledge support from the NIH (NS057198, EB00790, MH111359, and S10RR029050) and the Ministry of Education, Youth and Sports of the Czech Republic (CEITEC 2020, LQ1601). KK was supported by postdoctoral fellowships from the International Headache Society in 2014 and The Scientific and Technological Research Council of Turkey in 2015. MT was supported by postdoctoral fellowship from the German Research Foundation (DFG TH 2031/1).

### Conflict of interest statement

The authors declare that the research was conducted in the absence of any commercial or financial relationships that could be construed as a potential conflict of interest.

## Supplementary material

Supplemental USER GUIDE file 1 **Installation_readme.txt file, 4 zipped software packages (NNE2.zip, MCRInstaller.zip, hana_refs.tar.gz, hana_stk.tar.gz; downloaded from** http://nil.ucsd.edu/data/NNE/NNE2_HDbase_v1.0/).

Supplemental USER GUIDE file 2 **Installation readme**.
