# Supplementary material for: Neurovascular Network Explorer 2.0: A Database of 2-Photon Single-Vessel Diameter Measurements from Mouse SI Cortex in Response To Optogenetic Stimulation
Source: Front Neuroinform. 2017 Feb 1;11:4. doi: 10.3389/fninf.2017.00004 (PMC5285378; doi:10.3389/fninf.2017.00004)
Supplement: Supplemental USER GUIDE file 1 — Installation_readme.txt file, 4 zipped software packages (NNE2.zip, MCRInstaller.zip, hana_refs.tar.gz, hana_stk.tar.gz; downloaded from http://nil.ucsd.edu/data/NNE/NNE2_HDbase_v1.0/). [file DataSheet1.DOCX]

**User guide to “Neurovascular Network Explorer 2.0” GUI**

NNE 2.0 consists of 4 data query and visualization panels (Panel 1 called Main Panel, Panel 2, Panel 3 and Panel 4). The progression from general to specific data pruning occurs across the 4 panels. The user can select, manipulate, visualize, and export specific data of interest based on search parameters such as cortical depth, branching order, and baseline diameter as well as Subject and Tree IDs. Panels 1-3 display (1) dilation time-courses (stimulus-induced diameter change as a function of time), and (2) scatter plots of onset time, time-to-peak, peak amplitude, and baseline diameter as a function of cortical depth. Panel 4 displays (1) dilation time-courses, (2) a reference image showing a horizontal (XY) plane of the vasculature where the dilation was measured, and (3) a corresponding 3D image stack (a structural volume of vasculature composed of sequential horizontal (XY) image planes along the cortical depth axis (Z)).

**Panel 1**

**
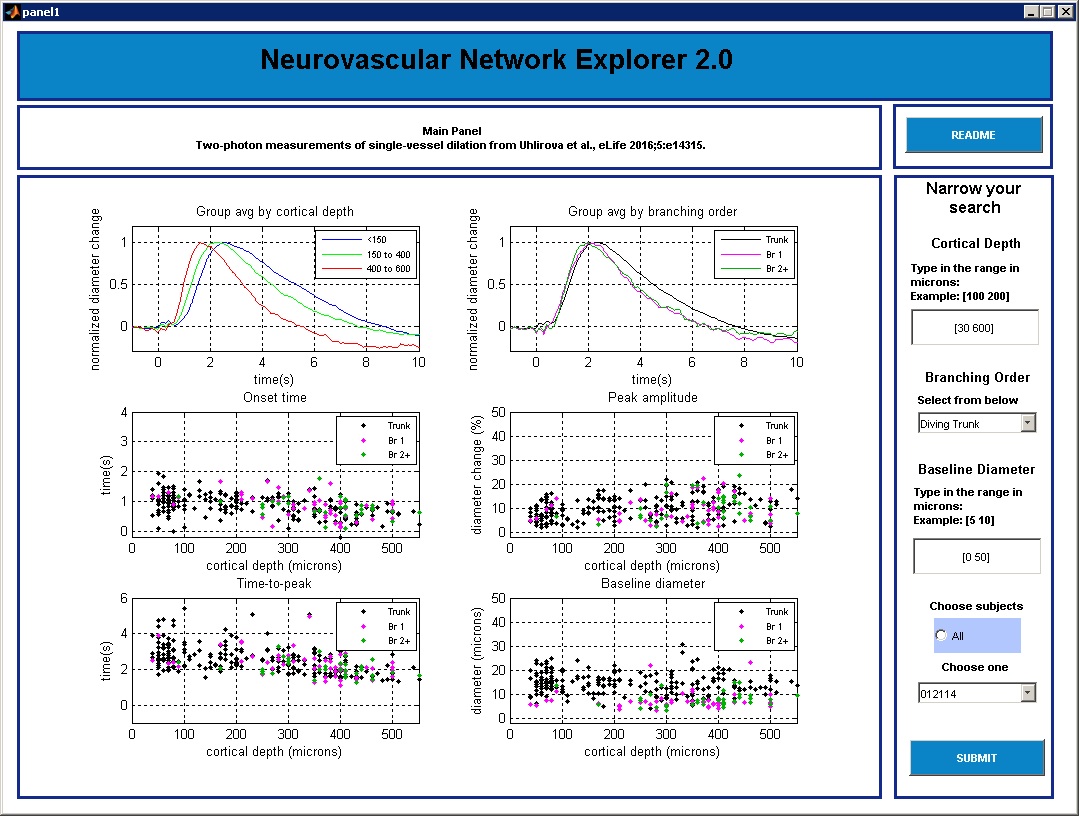
**

Panel 1 (Main Panel) consists of two primary sections: 1) the right column with 4 control/selection boxes allowing the user to interact with the database; 2) the left column for overview of the entire dataset.

On the right column, the user enters query parameters below the ‘README’ button as following:

1. [Cortical Depth]: Enter the range in microns as plain text within brackets, i.e., a cortical depth range between 200 and 400 microns below the cortical surface is [200 400].
2. [Branching Order]: Use the drop-down menu and select from one of 4 options: ‘Surface’ stands for surface arterioles, ‘Diving Trunk’, ‘First Order Branches’, ‘Higher Order Branches’. The default option is ‘Diving Trunk’. Please note that there are currently no entries for Surface Arterioles in the optogenetic database. This option is kept for future updates to the current database or for the users own databasing efforts.
3. [Baseline Diameter]: Enter the range in microns as plain text within brackets, i.e., a baseline diameter range between 5 and 20 microns is [5 20]. The maximum range of the data is 30-560 microns.
4. [Choose subjects]: [All] and [Choose one] are mutually exclusive. If [All] option is selected, it will ignore the value selected in the drop-down menu from [Choose one].
5. [Submit]: Once all selections are made, a click on the ‘SUBMIT’ button brings up Panel 2.

**Panel 2:**


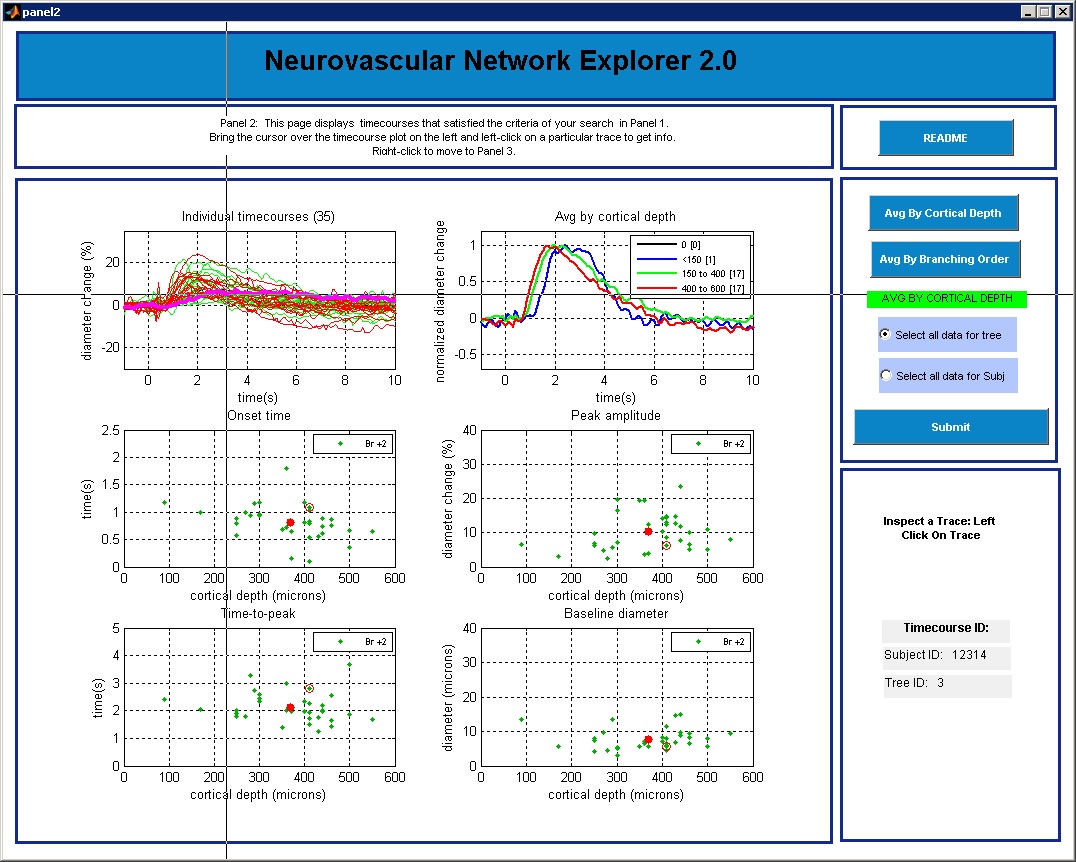


Panel 2 consists of 2 primary sections: 1) the right column allowing the user to interact with the database; 2) the left column for data visualization based on the selection specified in the right column and Panels 1 and 2.

1. The left column is initially blank. The user needs to choose in the column on the right whether to perform group average by cortical depth or branching order and click ‘SUBMIT’ button before 6 plots are populated on the left. The top left plot shows a family of overlaid individual time-courses that satisfy the search criteria specified on Panel 1. The top right plot shows peak-normalized averages. Four scatter plots below display the onset time, time-to-peak, peak amplitude, and baseline diameter as a function of the cortical depth. Larger red dot displays the average value for each scatter plot.
2. The user can select a particular entry by left clicking on a time-course in the top left plot. Subsequently, the time-course will be highlighted, along with the corresponding onset time, time-to-peak, peak amplitude, and baseline diameter in the other plots. In addition, the corresponding Subject and Tree IDs will be displayed in the lower right column.
3. The user can explore all entries associated with this Subject ID (all measurements within this individual subject) or Tree ID (all measurements along this specific arteriolar tree). The default option is ‘Select all data for tree’. Please note that this choice (made in the upper right column) needs to be done along with the choice of group average type before the initial clicking of “SUBMIT” button. A right click anywhere brings up Panel 3 that shows all individual time-courses for that Tree ID.
4. If inspection of all measurements within a subject is desired, the user needs to return to Panel 1 (by closing Panel 3 window [x]) where he repeats the selection of categories and after bringing up Panel 2 chooses ‘Select data for subject’ followed by a choice of trace and a right click anywhere.
5. Close this window [x] to go back to Panel 1.

**Panel 3:**


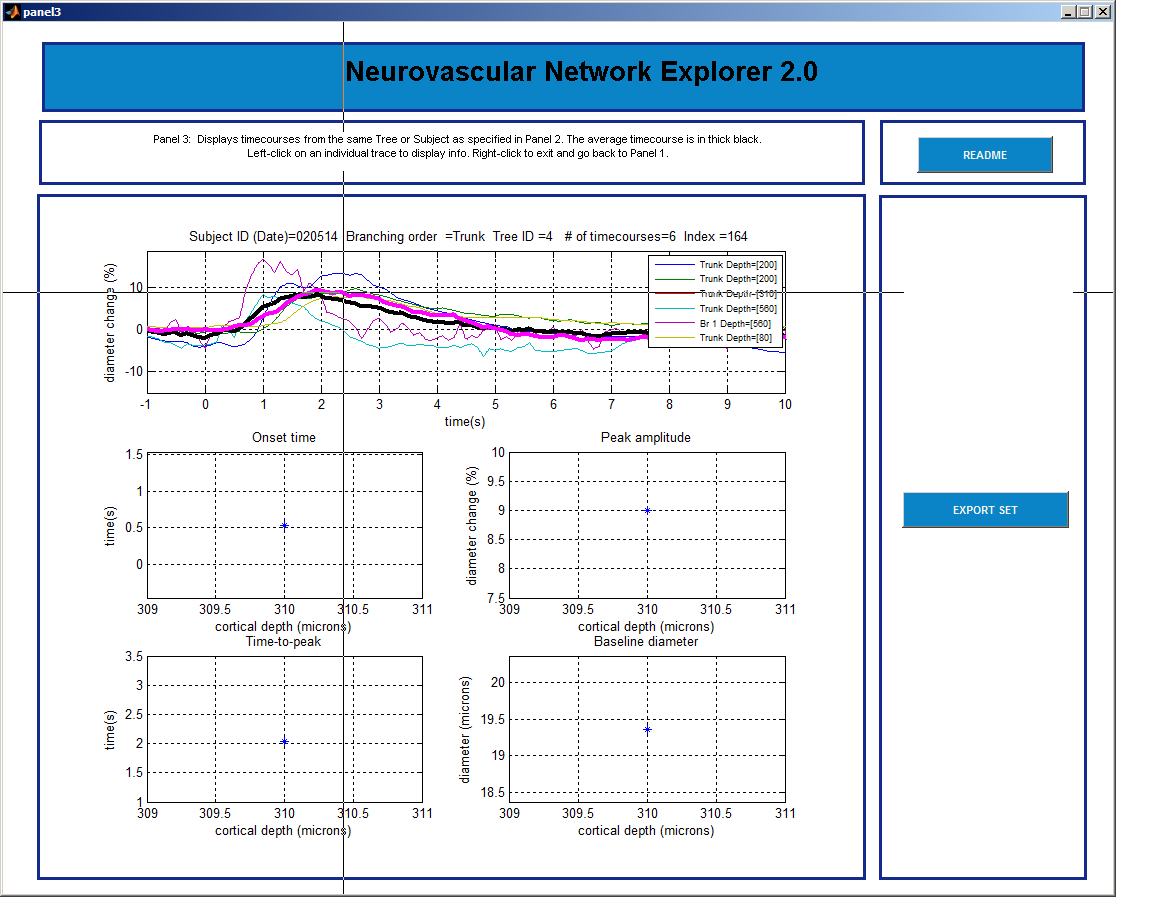


Panel 3 consists of two primary sections: 1) the left column displays data specified in Panel 2; 2) the right column allows the user to export the set of time-courses displayed in the left panel or continue to the next panel in order to view the reference image and a corresponding 3D image stack.

1. The left column consists of 5 graphs. The top graph shows all time-courses available for a single Subject ID or Tree ID specified in Panel 2. The information of ‘Subject ID’, ‘Tree ID’ and the ‘Number of time-courses’ are displayed above the graph. Right upon opening Panel 3 the ‘Tree ID’ relates to the time-course selected in Panel 2. Four scatter plots at the bottom of the left panel display the onset time, time-to-peak, peak amplitude, and baseline diameter as a function of the cortical depth for all time-courses in the top graph.
2. The user can select a particular entry by left clicking on the time-course in the top graph. Subsequently, the time-course will be highlighted and additionally an average time-course will be displayed in thick black. Four graphs below will display the onset time, time-to-peak, peak amplitude, and baseline diameter associated with the selected database entry. The related information of ‘Subject ID’, ’Branching order’, ‘Tree ID’, ‘Number of time-courses’ and ‘Index’ of the database entry (corresponding to the row number in the vdb_gnu matrix) will be displayed above the top graph.
3. Left click on [EXPORT SET] in the right column will export a family of time-courses that belong to the chosen Subject, or a family of time-courses that belong to the chosen Tree displayed in the left column, top panel. Three files will be saved in the same directory that NNE 2.0 is running from:

I. ‘vdb_subset.xls’ containing the time and dilation amplitude vectors along with their database indexes;

II. ‘vdb_subset.csv’ containing 2*n* vectors where *n* is the number of time-courses to be exported. The odd columns are the time vectors (in seconds) and the following even column is the corresponding dilation time-course.

III. ‘vdb_subset.mat’ containing a selected subset of entries from the vdb_gnu.mat. The descriptive parameters are in the following order: ‘Date’, ‘Index’, ‘Set’, ‘Time’, ‘Timecourse’, ‘Normalized timecourse’, ‘Branching order’, ‘Z-stack index’, ‘Time of peak’, ‘Ref image pointer’, ‘Depth’, ‘X-axis intercept’, ‘Baseline Diameter’, ‘Z-stack pointer’, ‘Peak Dilation amplitude’, ‘Ref image umpp’, ‘Z-stack umpp’, ‘Map pointer’, ’Z-stack step’.

The user must make sure any of the files are not currently open to allow NNE 2.0 overwriting the files if they exist.

1. Close this window [x] to go back to Panel 1.
2. If ‘Select all data for tree’ was chosen in Panel 2, a right click anywhere will open the next panel - Panel 4. Panel 4 will allow the user to browse through 3D image stacks and reference images associated with selected traces. Loading of Panel 4 is indicated in the right column on top (‘Loading Panel 4. Please Wait!’). An attempt to access Panel 4 following the option ‘Select all data for subject’ will result in restarting NNE 2.0 which will allow the user to select data for tree in Panel 2 and proceed to Panels 3 and 4.

**Panel 4:**


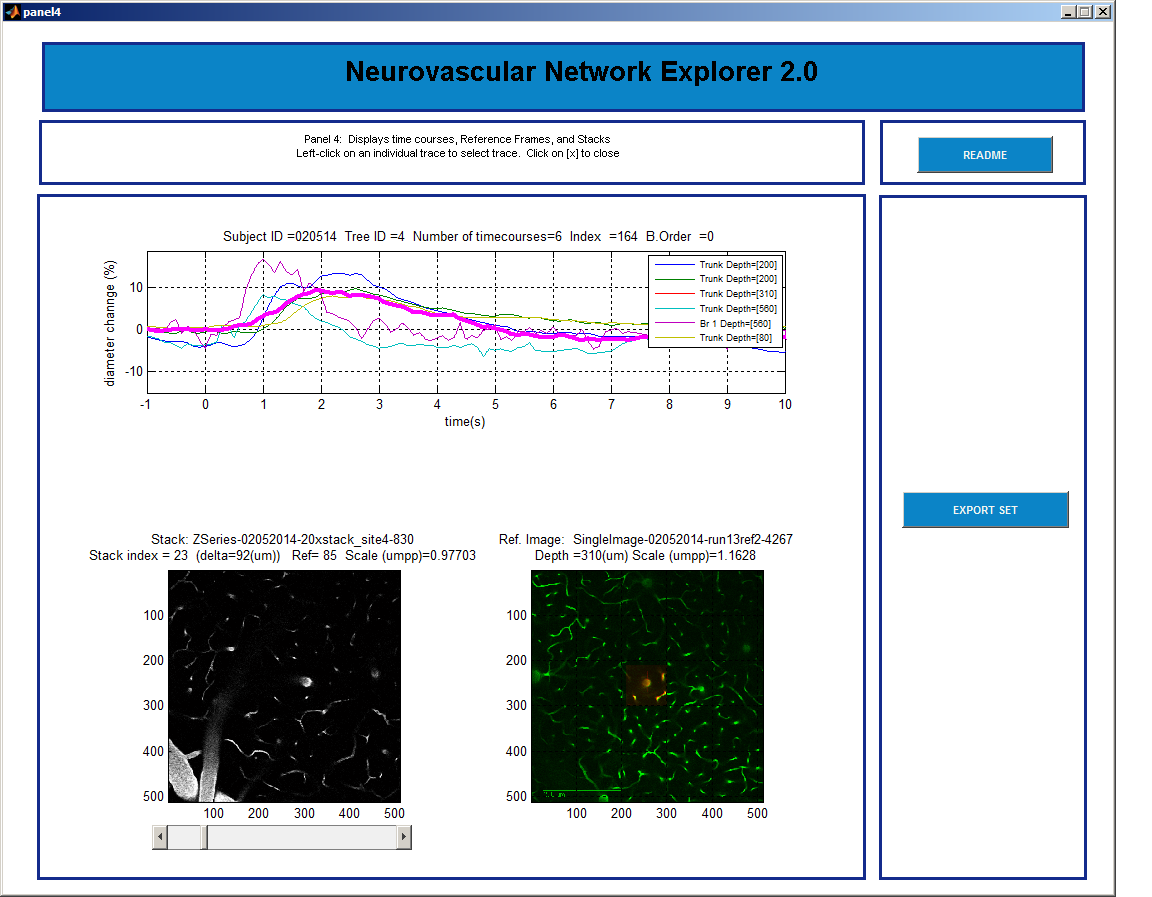


Panel 4 consists of two primary sections: 1) the left column displays data specified in Panel 2 and the corresponding reference images and 3D image stacks; 2) the right column serves for exporting data displayed in the left column.

1. The top graph in the left column is identical to the top graph in Panel 3 and shows all individual time-courses that satisfy the search criteria specified in Panel 2. The information of ‘Subject ID’, ‘Tree ID’, ‘Number of time-courses’, index number (‘Index’) and branching order (‘B. Order’) related to the chosen highlighted time-course are displayed above the graph. The user can select this or another entry by left clicking on the desired time-course trace and explore the associated reference image and the 3D image stack below. The reference image (bottom right) displays the XY image plane of the measured location. The vessel of interest lies within a red semi-transparent rectangle which is overlaid on top of the green reference image. The user can access the full-resolution reference image using the filename reference which is exported in ‘ref_stacks_trace.xls’ from Panel 4 or stored in vdb{i,16} when working in Matlab. The scanning path is marked as a red line across the vessel. If multiple vessels (e.g. a diving arteriole and its branches) were scanned simultaneously, the scanning line will cross all the measured vessels. In this case the branching order (‘B. Order’) on top of the time-courses plot defines which scanning line corresponds to the selected time-course. This can be understood either right from the reference image or from the structural 3D reconstruction of the vascular tree. Information of the reference image name (‘Ref. Image’) as well as the cortical ‘Depth’ in microns and ‘Scale’ in microns per pixel are displayed on top of the reference image. A note ‘NO REFERENCE IMAGE FOUND: index = ’ will appear when a reference image is missing. A 3D image stack for the selected entry can be explored in the image on the left. Initially the top image of the stack is displayed and the user can scroll through the stack using the slider or the arrows below the image. The information on top describe the image number within the stack (‘Stack index’), the vertical distance from the top frame in microns (‘delta’), the stack index for a frame that is the closest match to the reference image (‘Ref’), the scale of the individual frames in microns per pixel and the location of the folder with the stack images. When a stack image is chosen which corresponds to the reference image (i.e. ‘Stack index’ = ‘Ref’), the stack image will be highlighted and a note ‘*** Frame level’ will be displayed. In most cases, the plane of the cortical surface did not exactly match the XY imaging plane. As a result, image stacks are titled relative to the cortical surface. Therefore ‘delta’ at the frame level does not match exactly the ‘Depth’ of the reference image which is approximate and was entered manually by the researcher during the acquisition. To further refine estimation of the depth, one can resample the image stack to align the XY plane with the cortical surface using algorithms available elsewhere. If there is no 3D image stack available for a database entry or no reference image/stack match was found the image will appear blank with a note *NO STACK MATCH*. The scale for a reference image and the corresponding stack is not always the same and can be found in the vdb_gnu matrix (vdb_gnu{i, 21} and vdb_gnu{i, 22}, respectively. This happens when the 3D image stack was acquired with a different zoom than the reference image.
2. The selected time-course as well as the information about the corresponding stack and reference image can be exported by left clicking on ‘EXPORT SET’ in the right column. The exported file *‘*ref_stacks_trace.xls’ will be saved into the location where NNE 2.0 runs from. It contains the time vector and the dilation amplitude time-course, database index number (‘E2’), location of the 3D stack image folder (‘I3’), the index number of stack frame matched to the reference image (‘G3’) and the location of the reference image (‘I4’). The stack and reference image paths strings included in the exported data file can be used to access the stack and image data saved in the ‘hana_stk’ (for 3D image stacks) and ‘hana_refs’ (for the reference images) subdirectories where the NNE 2.0 is installed. Entries with no reference image will not be exported and the user will be prompted to choose a different trace.
3. Left click on the [x] will close Panel 4 and the user can go back to Panel 1.

**General notes:**

During the progression from Panel 1 to Panel 4, NNE 2.0 generates 3 files which are saved in the folder where it is running from. They are ‘vdbData.mat’, ‘curr_idx.mat’ and ‘ref_idx.mat’. Those files are for the internal use of the software and are not to be used or modified by the user.

**Notes for outliers:**

1. Out of 305 entries there are 9 entries which don’t have a reference image, 31 entries with a missing z-stack and 52 entries with no match of reference image to the z-stack frame.
2. Entries with index numbers 156-159 and 160-161 were measured along the same vascular tree although they have references to a different 3D stack (‘ZSeries-02052014-20xstack_site3_top-826’ and ‘ZSeries-02052014-20xstack_site3_bottom-825’, respectively). The ‘top’ stack scans the vascular tree from 0-432 μm and the ‘bottom’ stack captures the tree from 495-567 μm.
