# Supplementary material for: Neurovascular Network Explorer 2.0: A Database of 2-Photon Single-Vessel Diameter Measurements from Mouse SI Cortex in Response To Optogenetic Stimulation
Source: Front Neuroinform. 2017 Feb 1;11:4. doi: 10.3389/fninf.2017.00004 (PMC5285378; doi:10.3389/fninf.2017.00004)
Supplement: Supplemental USER GUIDE file 2 — Installation readme. [file DataSheet2.DOCX]

This folder contains the new version of the NNE for exploring neurovascular data.

Steps to take:

0) To unzip tar.gz files you need a decompression tool like winrar from rarlabs or other freeware to unzip tat.gz archives

1) Create a directory for your NNE system (Example C:\NNE). Download both compressed *.tar.gz files, the data base archives, and unzip them under NNE directory. That will create two data sub-directories under NNE\, namely \hana_stk and \hana_refs.

2) Download MCRInstaller.zip and unzip the file MCRInstaller.exe. This is the MATLAB run-time libraries.

3) Run MCRInstaller.exe after unzipping in step 2 to install MATLAB run-time libraries in your system

4) Download NNE2.zip and unzip all contents into your NNE directory

5) Run NNE2.exe from the NNE directory with Administrative privileges (To be able to save data export files)
